# Detection and Complete Genome Analysis of Circoviruses and Cycloviruses in the Small Indian Mongoose (*Urva auropunctata*): Identification of Novel Species

**DOI:** 10.3390/v13091700

**Published:** 2021-08-27

**Authors:** Kerry Gainor, Anne A. M. J. Becker, Yashpal S. Malik, Souvik Ghosh

**Affiliations:** 1 Department of Biomedical Sciences, Ross University School of Veterinary Medicine, Basseterre P.O. Box 334, Saint Kitts and Nevis, West Indies; KerryGainor@students.rossu.edu (K.G.); ABecker@rossvet.edu.kn (A.A.M.J.B.); 2 College of Animal Biotechnology, Guru Angad Dev Veterinary and Animal Science University, Ludhiana 141001, India; malikyps@gmail.com

**Keywords:** circovirus, cyclovirus, small Indian mongoose, complete genome analysis, novel species

## Abstract

Fecal samples from 76 of 83 apparently healthy small Indian mongooses (*Urva auropunctata*) were PCR positive with circovirus/cyclovirus pan-*rep* (replicase gene) primers. In this case, 30 samples yielded high quality partial rep sequences (~400 bp), of which 26 sequences shared maximum homology with cycloviruses from an arthropod, bats, humans or a sheep. Three sequences exhibited maximum identities with a bat circovirus, whilst a single sequence could not be assigned to either genus. Using inverse nested PCRs, the complete genomes of mongoose associated circoviruses (Mon-1, -29 and -66) and cycloviruses (Mon-20, -24, -32, -58, -60 and -62) were determined. Mon-1, -20, -24, -29, -32 and -66 shared <80% maximum genome-wide pairwise nucleotide sequence identities with circoviruses/cycloviruses from other animals/sources, and were assigned to novel circovirus, or cyclovirus species. Mon-58, -60 and -62 shared maximum pairwise identities of 79.90–80.20% with human and bat cycloviruses, which were borderline to the cut-off identity value for assigning novel cycloviral species. Despite high genetic diversity, the mongoose associated circoviruses/cycloviruses retained the various features that are conserved among members of the family *Circoviridae*, such as presence of the putative origin of replication (*ori*) in the 5′-intergenic region, conserved motifs in the putative replication-associated protein and an arginine rich region in the amino terminus of the putative capsid protein. Since only fecal samples were tested, and mongooses are polyphagous predators, we could not determine whether the mongoose associated circoviruses/cycloviruses were of dietary origin, or actually infected the host. To our knowledge, this is the first report on detection and complete genome analysis of circoviruses/cycloviruses in the small Indian mongoose, warranting further studies in other species of mongooses.

## 1. Introduction

Viruses belonging to the family *Circoviridae* (genera *Circovirus* and *Cyclovirus*) contain a covalently closed, circular, single-stranded DNA genome (~1.7–2.1 kb in size) [1,2]. The circovirus and cyclovirus genomes have an ambisense organization, consisting of at least two inversely arranged open reading frames (ORFs) that encode the replication-associated protein (Rep) and the capsid protein (Cp) [1,2]. In circoviruses, the ORF coding for the Rep is organized on the virion-sense (positive-sense) strand, whilst the cycloviral Rep is encoded by the complementary (anti-sense) strand of a double-stranded DNA replicative form. The Rep is the most conserved protein in circoviruses/cycloviruses and contains sequence motifs that are characteristic of proteins participating in rolling-circle replication (RCR) [1,2]. On the other hand, the Cp has been found to be significantly more divergent and is characterized by the presence of an arginine/basic amino acid (aa) rich region in the amino terminus that might be involved in DNA binding activity [1,2,3,4,5].

Circovirus and cyclovirus genomes contain two intergenic regions (IR) that are located between the initiation codons (5′-IR) and between the stop codons (3′-IR) of the *rep* and *cp* genes, although cycloviruses lacking the 3′-IR have also been reported [1,2,3,4,5]. The 5′-IR contains the origin of replication (*ori*), characterized by a conserved nonanucleotide motif (nAnTATTAC, where ‘n’ represents any nucleotide (nt)) at the apex of a stem-loop structure [1,2,6,7]. The 3′-IR is shorter than 5′-IR, and consistently smaller in cycloviruses than those observed in circoviruses [1,2,3,4,5].

Based on genome-wide pairwise nt sequence identities and phylogenetic analysis, the International Committee on Taxonomy of Viruses (ICTV) has recognized at least 49 and 52 species within the genera *Circovirus* and *Cyclovirus*, respectively [https://talk.ictvonline.org/ictv-reports/ictv_online_report/ssdna-viruses/w/circoviridae, accessed 10 July 2021]. Circoviruses and cycloviruses have been reported in various mammals, birds and arthropods [1,2,3,4,5,8,9,10,11,12,13,14,15,16,17,18,19,20,21,22,23]. Circoviruses have also been detected in fish and reptiles [2,23,24,25,26,27,28]. Despite identification in a wide variety of animals, only some circovirus species have been associated with clinical conditions [15,29,30,31,32,33,34,35], notable among which are porcine circovirus 2 associated diseases in pigs [17,29] and beak and feather disease virus associated psittacine beak and feather disease in wild and captive psittacine birds [30]. Furthermore, circovirus infection has been related to lymphopenia and immunosuppression [1,2]. On the other hand, since the current members of the genus *Cyclovirus* were discovered by molecular methods, their definitive host/s and pathogenesis are largely unknown [1,2,4], although cycloviruses have been detected in humans with paraplegia [36] and pneumonia [19]. The detection of many of the cycloviruses in the gut/fecal samples also indicate a possible origin from consumed food, or enteric parasites [1,2,4,21].

Mongooses are small terrestrial carnivores that belong to the family *Herpestidae* [37,38]. Due to their invasive and scavenging behavior, and proximity to humans and other animals, mongooses can serve as potential carriers of viral pathogens [37,39,40]. However, to date, limited virological studies have been conducted in mongoose populations. Mongooses are recognized as an important enzootic reservoir of the rabies virus, especially in the Caribbean region [39], and have been shown to be a reservoir animal for hepatitis E virus on Okinawa Island, Japan [40]. Other viruses reported in mongooses include *Carnivore protoparvovirus 1*, cowpox virus, feline panleukopenia virus, picobirnavirus and thogoto virus [41,42,43,44,45]. Among the eukaryotic circular Rep-encoding single-stranded DNA (CRESS DNA) viruses, four novel gemycircularviruses were detected in an Egyptian mongoose (*Herpestes ichneumon*) [46]. In the present study, we report for the first-time detection and complete genomic analysis of circoviruses and cycloviruses in the small Indian mongoose (*Urva auropunctata*).

## 2. Materials and Methods

### 2.1. Ethics Statement

This study received approval from the Institutional Animal Care and Use Committee (IACUC) of the Ross University School of Veterinary Medicine, St. Kitts and Nevis (IACUC protocol title: trapping and necropsy for mongoose microbial ecology study. Approved IACUC protocol number: 17.04.13, dated 13 April 2017).

### 2.2. Sample Collection

During April–July 2017, non-diarrheic fecal samples were obtained from the rectum and distal part of the colon of 83 small Indian mongooses that were trapped, euthanized and necropsied under sterile conditions for a gut microbiome study on the Caribbean island of St. Kitts [47]. The samples were kept at −80 °C until further analyses.

### 2.3. Amplification of Viral DNA

Viral DNA was extracted from the fecal samples using the QIAamp Fast DNA Stool Mini Kit (Qiagen Sciences, Germantown, MD, USA) following the instructions provided by the manufacturer. The samples were screened for the presence of circoviruses and cycloviruses by nested PCR assays using pan-*rep* primers (primers CV-F1, CV-R1, CV-F2 and CV-R2, targeting a short stretch (~400 bp) of the Rep-encoding ORF), as described previously [3]. Additional primers were designed from the partial Rep-encoding ORF sequences and used in inverse nested PCRs to amplify the complete genomes of the mongoose associated circovirus and cyclovirus (Supplementary material S1). PCRs were performed using the Platinum™ Taq DNA Polymerase (Invitrogen™, Thermo Fisher Scientific Corporation, Waltham, MA, USA) according to the manufacturer’s instructions. Sterile water was used as a negative control in all PCR reactions.

### 2.4. Nucleotide Sequencing

The PCR amplicons were purified using the Wizard^®^ SV Gel and PCR Clean-Up kit (Promega, Madison, WI, USA) following the instructions provided by the manufacturer. Nucleotide sequences were obtained using the ABI Prism Big Dye Terminator Cycle Sequencing Ready Reaction Kit (Applied Biosystems, Foster City, CA, USA) on an ABI 3730XL Genetic Analyzer (Applied Biosystems, Foster City, CA, USA).

### 2.5. Sequence Analysis

Homology search for related nt and deduced aa sequences were performed using the standard BLASTN and BLASTP program (Basic Local Alignment Search Tool, www.ncbi.nlm.nih.gov/blast, accessed on 22 June 2021), respectively. Putative ORFs encoding the viral Rep and Cp were identified using the ORF finder (https://www.ncbi.nlm.nih.gov/orffinder/, accessed on 20 June 2021), whilst those with a putative intron between the Rep coding sequences (CDS) were determined by BLASTN analysis with the CDS feature. Pairwise sequence (%) identities for the complete viral genomes, and the putative Rep and Cp were determined using the MUSCLE algorithm in the SDTv1.2 program, as described previously [2,48]. On the other hand, pairwise identities between the partial Rep-encoding ORF sequences were calculated using the MUSCLE alignment program (https://www.ebi.ac.uk/Tools/msa/muscle/, accessed on 23 June 2021) and the ‘align two or more sequences’ option of BLASTN program (https://blast.ncbi.nlm.nih.gov/, accessed on 23 June 2021). The maps of the circular viral genomes were constructed with the ‘Draw Custom Plasmid Map’ program (https://www.rf-cloning.org/savvy.php, accessed on 20 June 2021). The putative stem-loop structure was identified in the viral genome using the mFold program [49].

Multiple alignments of nt and deduced aa sequences were carried out using the MUSCLE algorithm embedded in the MEGA7 software [50]. Phylogenetic analysis was performed by the maximum likelihood (ML) method using the MEGA7 software [50], with the GTR+G model of substitution and 1000 bootstrap replicates, as described previously [2]. The complete genomes of the mongoose associated circoviruses and cycloviruses were investigated for recombination events using the RDP4 program with default parameters [51]. A circovirus/cyclovirus sequence was determined as a recombinant if it was supported by two, or more than two detection methods (3Seq, BOOTSCAN, CHIMERA, GENECONV, MAXCHI, RDP and SISCAN) with a highest acceptable *p*-value of *p* < 0.01 with Bonferroni’s correction [17,51].

### 2.6. GenBank Accession Numbers

The GenBank accession numbers for the mongoose associated CRESS DNA viral sequences determined in this study are MZ382570-MZ382599.

## 3. Results and Discussion

### 3.1. Detection of Circoviruses and Cycloviruses in the Small Indian Mongoose

**Figure 1:**
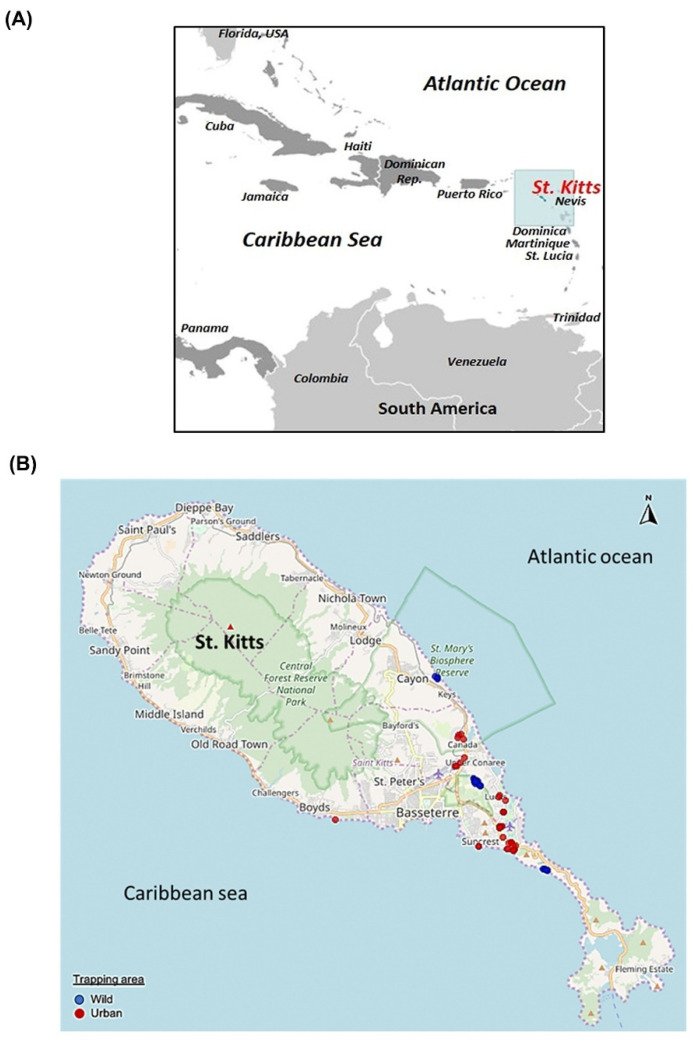
(**A**) Geographical location of the Caribbean island of St. Kitts. The map in Figure 1A was obtained from https://www.cia.gov/library/publications/the-world-factbook (accessed on 1 April 2021). (**B**) Map of St. Kitts showing the mongoose trapping sites. The trapping sites in the wild and urban habitats are shown with blue and red, respectively. The figure originally appeared in Kleymann et al., 2020 [44], and was used here with permission from the corresponding author of the publication [44].

The small island of St. Kitts (~69 square miles, human population of ~35,000) is inhabited by a large population of the small Indian mongoose (~45,000) that dwell in wild and urban habitats (Figure 1A,B) [44,52], [https://www.sknbs.org/about-bureau/about-st-kitts-and-nevis/, accessed on 14 July 2021]. In the present study, single fecal samples from 76 (91.56%) of the 83 small Indian mongooses yielded the expected ~400 bp amplicon with circovirus/cyclovirus pan-*rep* primers in screening PCR assays. In this case, 39 of the 76 positive samples showed strong PCR amplification and were sequenced for the partial *rep* gene. By BLASTN analysis, all the mongoose-associated partial CRESS DNA viral sequences shared maximum homology with published circovirus, or cyclovirus *rep* gene sequences, except for Mon-66, which shared maximum pairwise nt sequence identities of 63.37% with that of an unclassified CRESS DNA virus (GenBank accession number KY487932) from a wastewater sample (Table 1). However, based on analysis of the complete genome sequence, Mon-66 was assigned to the genus *Circovirus* (Table 2). Nine of the partial *rep* sequences lacked high quality and were excluded from further analysis.

Based on BLASTN analysis and pairwise nt sequence identities of partial *rep*, the mongoose associated partial CRESS DNA viral sequences were classified into at least 8 putative groups (designated as I-VIII) (Table 1). Mongoose associated partial *rep* sequences sharing >95% pairwise identities between themselves were assigned to the same group (Table 1). Group-I sequences shared maximum pairwise identities of 77.65–79.20% with circovirus isolate C072 that was detected in the intestinal sample from a vesper bat (*Myotis fimbriatus*) in China (Table 1). Group II-VII exhibited maximum homology with cyclovirus *rep* sequences (Table 1). Group-II and -VI sequences shared maximum homology (pairwise identities of 97.62–98.31% and 77.47–82.21%, respectively) with cycloviruses from bats (Table 1). Group-III and -V consisted of a single *rep* sequence each, and shared maximum pairwise identities of 94.25% and 93.11% with a cockroach associated cyclovirus and a sheep associated cyclovirus, respectively (Table 1). Group-IV was the largest, consisting of 15 partial *rep* sequences that shared maximum pairwise identities of 95.37–97.48% with human cyclovirus TN2 (detected in fecal sample of a healthy child who came in contact with a non-polio-infected acute flaccid paralysis patient [3]). Group-VII sequences shared maximum homology (pairwise identities of 91.84–92.07%) with human cyclovirus VS5700009 that was detected in the serum of a patient with unexplained paraplegia [36].

Even though we reported high rates of detection of CRESS DNA viral sequences that exhibited maximum homology with circoviruses, or cycloviruses, all the fecal samples were obtained from apparently healthy mongooses, indicating a lack of association between these viruses and clinical conditions. Circoviruses appear to primarily infect vertebrates, whilst those detected in hematophagous arthropod vectors might actually be viruses of mammals, or birds that these insects feed upon [2,5,21,23]. On the other hand, cycloviruses have been reported in a wide array of both invertebrates and mammals [2,5,21,23]. Based on these observations, it has been proposed that cycloviruses are more diverse and widespread than circoviruses [2,5,21,23]. In the present study, ~90% (35/39) of the partial *rep* sequences shared maximum pairwise identities with cycloviruses, which constituted 6 of the 8 putative groups of diverse CRESS DNA viral sequences (Table 1). Since only fecal samples were analyzed, and the small Indian mongoose has been known to feed on small mammals, reptiles, birds, bird and reptile eggs, crustaceans, insects and human waste [53], we could not determine whether the mongoose associated CRESS DNA viruses replicated in the host or were of dietary origin.

Based on the detection of closely related circoviruses/cycloviruses in different animal species, especially in tissues, some studies have proposed interspecies transmission events within the family *Circoviridae* [2,3,5,54]. In this study, 15 (group-IV) and 2 (group-II) of the mongoose associated partial *rep* sequences were closely related (>95% pairwise nt identities) to cycloviruses from humans and bats, respectively (Table 1). St. Kitts island has a sizeable bat population [55], and the small Indian mongoose has often been seen in close proximity to humans [44], offering an ideal environment for interspecies transmission events. However, we could not obtain the full-length genome sequences for group-II viruses, whilst that of complete genome of human cyclovirus TN2 (closely related to group-IV sequences) was not available in the GenBank database. Furthermore, caution should be exercised whilst commenting on cross-species transmission of circoviruses/cycloviruses from fecal samples, as they may have a dietary origin [2,3,5,54], especially in mongooses that have a wide-range of feeding habits [53]. Interestingly, one of the mongoose associated partial *rep* sequences (group-III) shared maximum homology with a cockroach associated cyclovirus, corroborating previous observations that cyclovirus sequences detected in vertebrate samples might be actually those from viruses of arthropods [21,23] (Table 1).

### 3.2. Analysis of the Complete Genomes of Mongoose Associated Circoviruses and Cycloviruses

**Figure 2:**
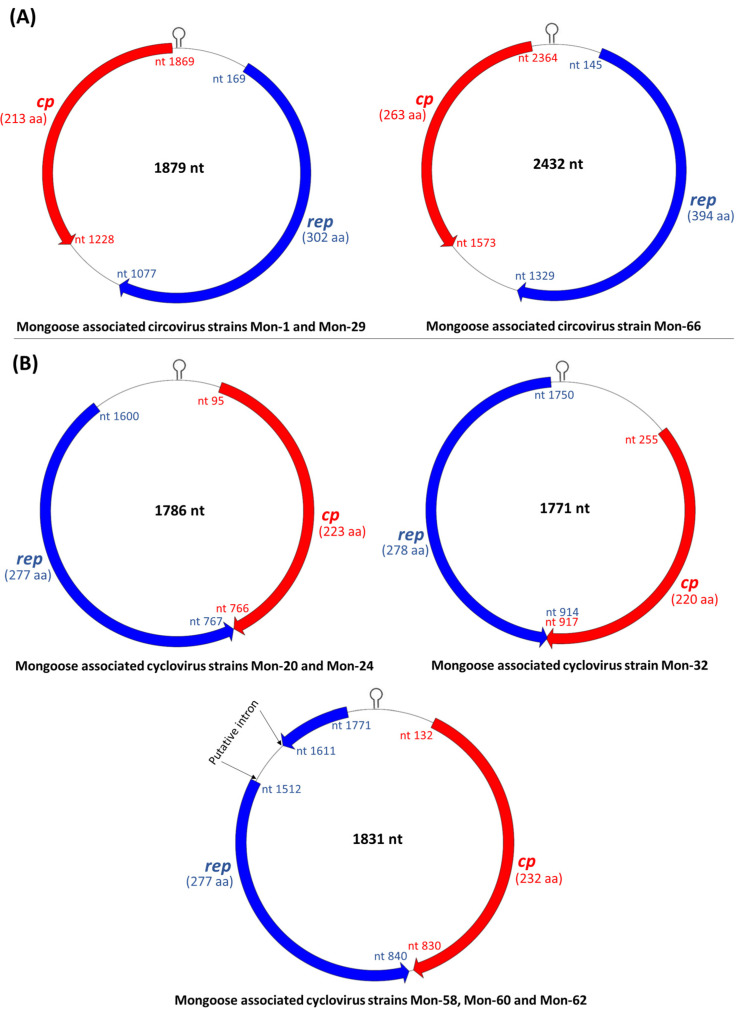
Genome organization of the mongoose associated circoviruses (**A**) and cycloviruses (**B**). The inversely arranged major open reading frames encoding the putative replication associated (Rep) and capsid (Cp) proteins are shown with blue and red arrows, respectively. The putative origin of replication (ori) characterized by a nonanucleotide motif at the apex of a stem-loop structure is marked in the 5′-intergenic region. The size of the Rep and Cp are shown in parenthesis. nt: nucleotide; aa: amino acid.

**Figure 3:**
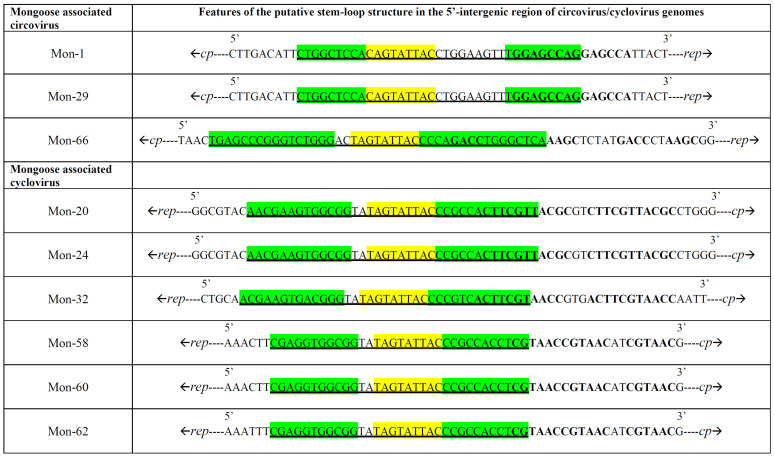
Potential stem-loop structures found in the 5′-intergenic region of mongoose associated circovirus and cyclovirus genomes. The putative stem-loop sequence is underlined. The nonanucleotide motif is shown with yellow. The complementary regions of the nucleotide sequence constituting the ‘putative stem’ are highlighted with green. The tandem repeat motifs are shown with bold font. *rep*: replicase gene; *cp*: capsid gene.

**Figure 4:**
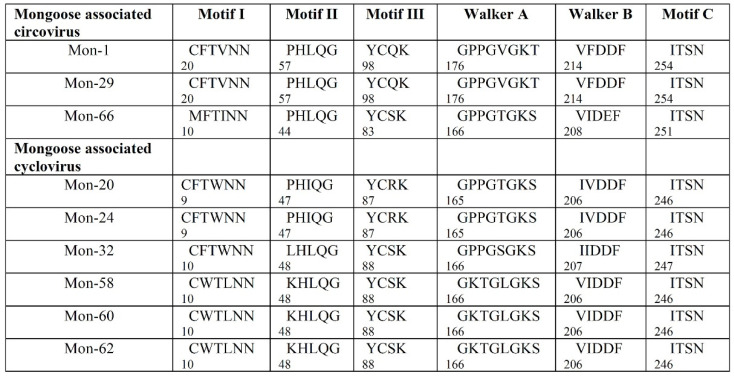
Presence of the conserved rolling circle replication (motifs I through III) and superfamily 3 helicase (Walker A and B, and motif C) motifs in the putative replication-associated proteins (Rep) of mongoose associated circovirus and cyclovirus. The number below the motif sequence indicates the position of the amino acid residue in the respective putative Rep protein.

Since the ICTV classification scheme for the family *Circoviridae* is based on genome-wide pairwise identities [1,2], attempts were made to determine the complete genome sequences of mongoose associated CRESS DNA viruses representing the putative groups I-VIII (Table 1). Using an inverse nested PCR assay, we obtained the full-length genome sequences of 2 group-I (Mon-1 and -29), a single group-IV (Mon-32), 2 group-VI (Mon-20 and -24), 3 group-VII (Mon-58, -60 and -62) and a single group-VIII (Mon-66) viruses (Table 1 and Table 2), whilst the complete genomes of group-II, -III and -V viruses could not be amplified. The genomic organization of the mongoose associated circoviruses and cycloviruses are shown in Figure 2. The complete genome sequences of the mongoose associated circoviruses and cycloviruses (collectively referred to as the ‘Mon sequences’) retained the various features that are conserved in members of the genera *Circovirus* and *Cyclovirus*, respectively, within the family *Circoviridae* (Figure 2, Figure 3 and Figure 4; Supplementary material S2) [1,2].

**Figure 5:**
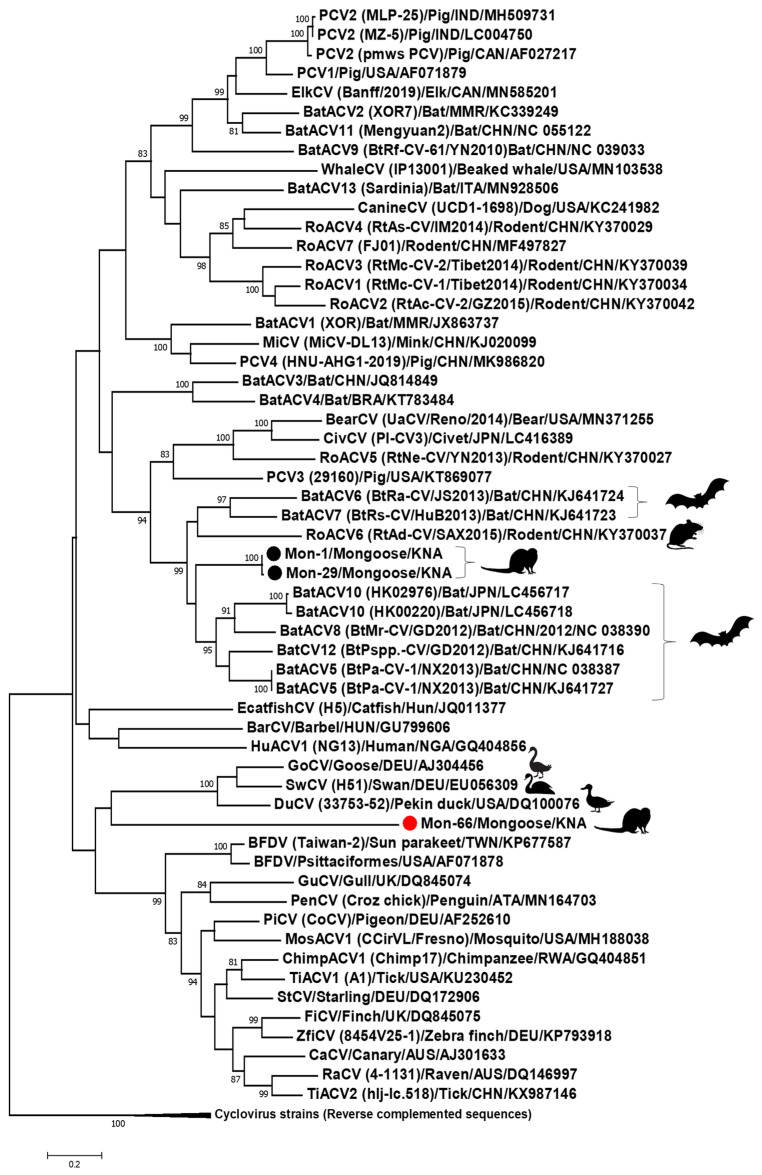
Phylogenetic analysis of the complete genomes of mongoose associated circoviruses (Mon-1, -29 and -66) with those of other circoviruses. The virus name/source (detected in animal species)/country are shown for the Mon sequences, while the species, or virus name (isolate)/source (detected in animal species)/country/GenBank accession number have been mentioned for the other circovirus sequences. The tree was constructed by the maximum likelihood (ML) method, with the GTR+G model of substitution and 1000 bootstrap replicates and rooted after using cyclovirus reverse complemented sequences as the outgroup. Scale bar, 0.2 substitutions per nucleotide. Bootstrap values of <80 is not shown. Mon-1 and -29 are shown with black circles, whilst Mon-66 is highlighted with a red circle. BarCV: barbel circovirus; BatACV: bat associated circovirus; BFDV: beak and feather disease virus; CaCV: canary circovirus; ChimpACV: chimpanzee associated circovirus; CivCV: civet circovirus; CV: circovirus; DuCV: duck circovirus; EcatfishCV: European catfish circovirus; ElkCV: elk circovirus; FiCV: finch circovirus; GoCV: goose circovirus; GuCV: gull circovirus; HuACV: human associated circovirus; MiCV: mink circovirus; MosACV: mosquito associated circovirus; PenCV: penguin circovirus; PCV: porcine circovirus; PiCV: pigeon circovirus; RaCV: raven circovirus; RoACV: rodent associated circovirus; StCV: starling circovirus; SwCV: swan circovirus; TiACV: tick associated circovirus; ZfiCV: zebra fish circovirus.

**Figure 6:**
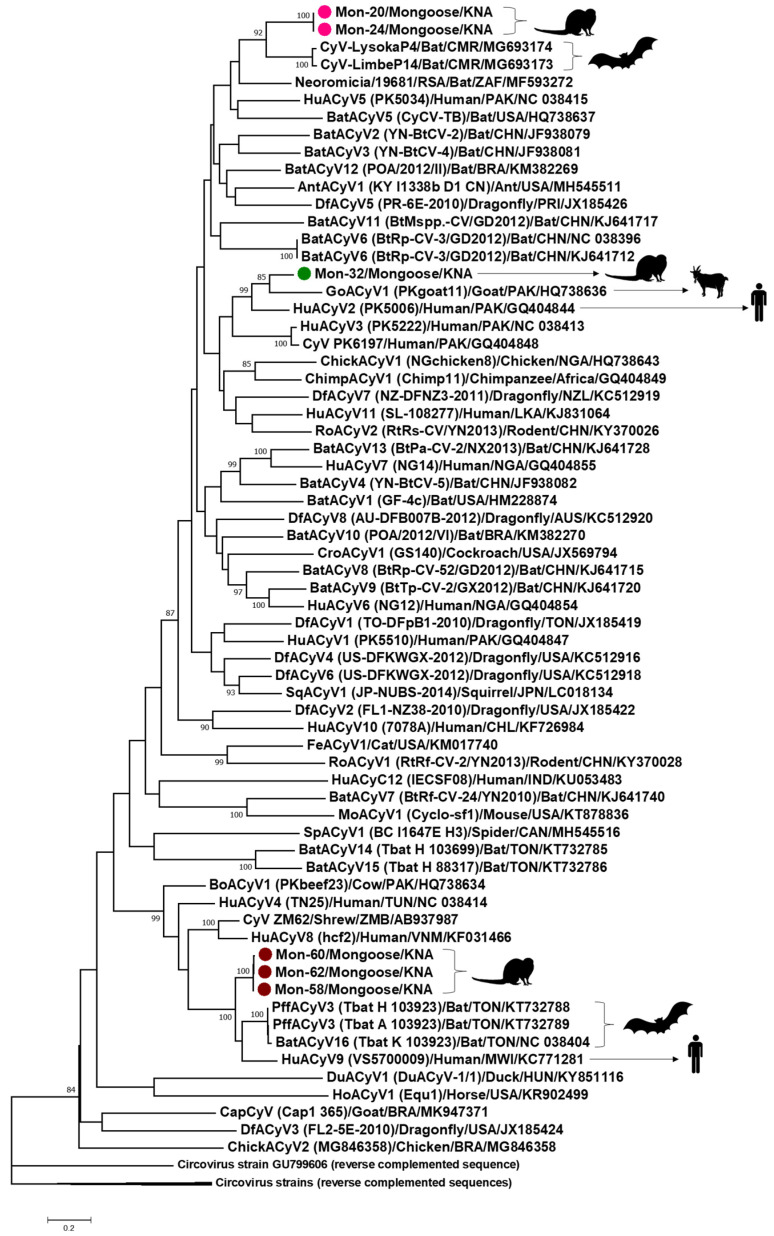
Phylogenetic analysis of the complete genomes of mongoose associated cycloviruses (Mon-20, -24, -32, -58, -60 and -62) with those of other cycloviruses. The virus name/source (detected in animal species)/country are shown for the Mon sequences, while the species, or virus name (isolate)/source (detected in animal species)/country/GenBank accession number have been mentioned for the other cyclovirus sequences. The tree was constructed by the maximum likelihood (ML) method, with the GTR+G model of substitution and 1000 bootstrap replicates and rooted after using circovirus reverse complemented sequences as the outgroup. Scale bar, 0.2 substitutions per nucleotide. Bootstrap values of <80 are not shown. Mon-20 and -24 are shown with pink circles, Mon-32 is highlighted with a green circle, whilst Mon-58, -60 and -62 are indicated with brown circles. AntACyV: ant associated cylcovirus; BatACyV: bat associated cyclovirus; BoACyV: bovine associated cyclovirus; ChickACyV: chicken associated cyclovirus; ChimpACyV: chimpanzee associated cyclovirus; CroACyV: cockroach associated cyclovirus; CyV: cyclovirus; DfACyV: dragonfly associated cycovirus; DuACyV: duck associated cyclovirus; FeACyV: feline associated cyclovirus; GoACyV: goat associated cyclovirus; HoACyV: horse associated cyclovirus; HuACyV: human associated cyclovirus; MoACyV: mouse associated cyclovirus; PffACyV: Pacific flying fox associated cyclovirus; RoACyV: rodent associated cyclovirus; SpACyV: spider associated cyclovirus; SqACyV: squirrel associated cyclovirus.

All the Mon sequences contained the putative *ori* in the 5′-IR, marked by the presence of the conserved nonanucleotide motif ((C/T)AGTATTAC) at the apex of a potential stem-loop structure (Figure 2 and Figure 3). Following ICTV guidelines [1,2], the first nt of the nonanucleotide motif was considered as ‘position one’ of the Mon sequences. Mon-1, -29 and -66 contained the putative *ori* in the Rep-encoding strand, whilst the putative *ori* was located in the Cp coding strand of Mon-20, -24, -32, -58, -60 and -62 (Figure 2). The 3′-IR was absent, or smaller in Mon-20, -24, -32, -58, -60 and -62 than those observed in Mon-1, -29 and -66 (Figure 2). Based on these observations, genome-wide pairwise identities, and phylogenetic analysis, Mon-1, -29 and -66 were classified as circoviruses, whilst Mon-20, -24, -32, -58, -60 and -62 were assigned to the genus *Cyclovirus* (Figure 2, Figure 3, Figure 4, Figure 5, Figure 6; Table 2, Table 3, Table 4; Supplementary Materials S2–S5).

Corroborating previous observations [1,2], the putative Rep of the mongoose associated circoviruses and cycloviruses retained the conserved RCR (motifs I through III) and superfamily 3 helicase (Walker A and B, and motif C) motifs (Figure 4). On the other hand, the putative Cp, although much more divergent than the Rep (Table 3 and Table 4), contained the conserved arginine rich region at the amino terminus, with the exception of Mon-66 (Supplementary material S2). Since recombinants have been reported in both circoviruses and cycloviruses [4,17,29,56,57,58], the Mon sequences were evaluated for potential recombination using the RDP4 program. However, we did not obtain reliable evidence for recombination events in the Mon sequences, except for Mon-32, which was identified by several models in the RDP4 program as minor parent to goat associated cyclovirus 1 (isolate PKgoat11, GenBank accession number HQ738636), whilst the major parent was unknown (Supplementary material S6).

The complete genomes of mongoose associated circoviruses Mon-1 and -29 were 1879 bp in size (Figure 2), which was comparable to those observed in most circoviruses [2]. Mon-1 and -29 were closely related to each other (Figure 5; Table 2, Table 3, Table 4; Supplementary Materials S3), and shared maximum pairwise identities of 67.40% and 67.20%, respectively, with that of bat circovirus isolate BtPspp.-CV (GenBank accession number KJ641716) from China [59], followed by identities of 66.90% and 66.60%, respectively, with bat associated circovirus 10 (isolate HK02976, GenBank accession number LC456717) from Japan (Table 2) [60]. Phylogenetically, the complete genome sequences of Mon-1 and -29 formed a distinct cluster within a clade that mostly consisted of circoviruses from bats, including isolates BtPspp.-CV and HK02976 (Figure 5). These observations were corroborated by analysis of the putative proteins (pairwise identities of Rep and Cp, and phylogenetic analysis of Rep) of Mon-1 and -29 (Table 3 and Table 4; Supplementary material S5).

The complete genome of Mon-66 was 2432 nt long (Figure 2), which was larger than those of most circoviruses [1,2]. Even though the partial *rep* sequence of Mon-66 exhibited maximum homology with that of an unclassified CRESS DNA virus (Table 1), the complete genome of Mon-66 shared maximum pairwise identities of 60.70% with that of porcine circovirus 2 isolate MZ-5 (GenBank accession number LC004750) from India (Table 2). Since the genome-wide pairwise identity cut-off value for assigning a sequence to a genus within the family *Circoviridae* is 55% [1,2], Mon-66 was classified as a circovirus. On the other hand, the Rep and Cp of Mon-66 shared maximum deduced aa identities of 51.20% and 44.40% with that of unclassified CRESS DNA viruses from a wild bird (MW182878) and a wastewater sample (KY487977), respectively (Table 3 and Table 4). Phylogenetically, Mon-66 formed an isolated branch within the clade of circoviruses (Figure 5; Supplementary material S5).

The complete genomes of the mongoose associated cycloviruses were 1771 nt, 1786 nt or 1831 nt long (Figure 2), which was within the size-range for cyclovirus genomes [1,2]. Mon-20 and -24 were fully identical in their complete genome sequences, and shared maximum homology (pairwise identities of 72.10%) with that of bat cyclovirus isolate CyV-LysokaP4 (GenBank accession number MG693174) from Cameroon (Table 2; Figure 6) [61]. Pairwise identities of the Rep and Cp, and phylogenetic analysis of Rep of Mon-20 and -24 also revealed similar findings (Table 3 and Table 4; Supplementary material S5).

Mon-32 exhibited maximum pairwise identity of 77.30% with the complete genome of goat cyclovirus isolate PKgoat11, followed by 75.30% with that of human cyclovirus isolate PK5006 (GQ404844) (Table 2). Cyclovirus isolates PKgoat11 and PK5006 were detected in the same study from Pakistan [3]. The Rep and Cp of Mon-32 exhibited maximum pairwise deduced aa identity of 87.10% and 51.60% with that of isolates PKgoat11 and PK5006, respectively (Table 3 and Table 4). Phylogenetically, Mon-32 clustered with isolate PKgoat11 within a clade that also consisted of PK5006 (Figure 6; Supplementary material S5).

The Rep-encoding ORF of Mon-58, -60 and -62 was interrupted by a putative intron (nt 1610–nt 1511) with a canonical splice donor site (GT) and splice acceptor site (AG) (Supplementary material S7). The complete genomes of Mon-58, -60 and -62 shared 99.30–99.50% pairwise identities between themselves, and maximum identities of 79.90–80.20% with those of human cyclovirus isolate VS570000 (GenBank accession number KC771281) from the CSF of a paraplegic patient in Malawi [36] and Pacific flying fox associated cyclovirus-3 isolate Tbat_H_103923 (KT732788) from the feces of a bat in Tonga (Table 2) [62]. Mon-58, -60 and -62 shared maximum pairwise identities of 92.40% with the Rep of isolate VS570000 (Table 3), and 56.10–56.60% with the Cp of isolate Tbat_H_103923 (Table 4). By phylogenetic analysis, Mon-58, -60 and -62 clustered together near bat associated cycloviruses (including isolate Tbat_H_103923) [62] and the human cyclovirus isolate VS570000 (Figure 6; Supplementary material S5) [36].

The ICTV has recommended a species demarcation threshold of 80% genome-wide pairwise nt sequence identity for members of the family *Circoviridae* [1,2]. Based on the ICTV classification system, mongoose associated circoviruses Mon-1/Mon-29 and Mon-66 qualify as novel species within the genus *Circovirus*, whilst mongoose associated cycloviruses Mon-20/Mon-24 and Mon-32 represent new species in the genus *Cyclovirus* (Table 2; Supplementary Materials S3 and S4). On the other hand, the maximum pairwise identities (79.90–80.20%) of mongoose associated cycloviruses Mon-58, -60 and -62 with cycloviruses from other animals/sources were borderline to the cut-off identity value for assigning novel cycloviral species (Table 2; Supplementary material S4).

### 3.3. Conclusions

Taken together, our findings suggest that CRESS DNA viruses are widely circulating in the small Indian mongoose population on the island of St. Kitts. However, in the absence of sampling from tissues, and considering that mongooses are polyphagous predators, we could not determine whether the circoviruses/cycloviruses in fecal samples of apparently healthy mongooses were of dietary origin, or actually infected the host. Based on the classification scheme proposed by the ICTV *Circoviridae* study group [1,2], we identified 2 novel species in each of the genera *Circovirus* and *Cyclovirus*, further expanding the genetic diversity of these viruses. However, despite high genetic diversity, the mongoose associated circoviruses/cycloviruses retained the various features that are conserved among members of the family *Circoviridae*. Studies aimed at detection of viral DNA in tissues, screening for virus-specific antibodies, in-vitro replication of virus in mongoose cells and virus inoculation in gnotobiotic animals are required to gain a proper understanding of circovirus/cyclovirus infection in mongooses. To our knowledge, this is the first report on detection and complete genome analysis of circoviruses and cycloviruses in the small Indian mongoose, warranting further studies in other species of mongooses.

## Figures and Tables

**Table 1:** Pairwise identities of partial circular Rep-encoding single-stranded (CRESS) DNA viral sequences detected in the small Indian mongoose (*Urva auropunctata*) with those reported from other animal species/environmental samples. Based on BLASTN analysis and pairwise nucleotide (nt) sequence identities, the mongoose associated partial CRESS DNA viral sequences were classified into at least 8 putative groups (designated as I–VIII). Mongoose associated viral sequences sharing >95% nt sequence identities between themselves were assigned to the same group and are highlighted with the same color.

CRESS DNA Viral Sequence from the Small Indian Mongoose	Putative Group	Length of High Quality nt Sequence Analyzed ^1^	GenBank Accession Number	Maximum Pairwise nt Sequence (%) Identity with Cognate CRESS Viral DNA Sequence (Virus Name/Detected in Animal, or Environment/Country/Year of Detection/GenBank Accession Number) from Other Animal Species, or Environment ^2^
Mon-1 ^3^	I	358 nt	MZ382570	77.65% with Bat circovirus isolate C072/Bat/China/2015/KX834490 ^3^
Mon-2	II	463 nt	MZ382579	97.62% with Pacific flying fox associated cyclovirus-3/Bat/Tonga/2015/KT732789
Mon-3	III	364 nt	MZ382580	94.25% with Cockroach-associated cyclovirus/Palmetto bug/USA/2011/JX569794
Mon-6	IV	363 nt	MZ382581	96.69% with Cyclovirus TN2/Human/Tunisia/2007/GQ404904
Mon-10	IV	390 nt	MZ382582	97.18% with Cyclovirus TN2/Human/Tunisia/2005/GQ404904
Mon-14	IV	381 nt	MZ382583	97.38% with Cyclovirus TN2/Human/Tunisia/2005/GQ404904
Mon-16	V	363 nt	MZ382584	93.11% with Cyclovirus NG_sheep50/Sheep/Nigeria/2009/GQ404982
Mon-18	IV	324 nt	MZ382585	95.37% with Cyclovirus TN2/Human/Tunisia/2005/GQ404904
Mon-20 ^3^	VI	364 nt	MZ382573	77.47% with Bat cyclovirus isolate CyV-LimbeP14/Bat/Cameroon/2013/MG693173 ^3^
Mon-22	IV	343 nt	MZ382586	96.79% with Cyclovirus TN2/Human/Tunisia/2005/GQ404904
Mon-24 ^3^	VI	364 nt	MZ382574	77.47% with Bat cyclovirus isolate CyV-LimbeP14/Bat/Cameroon/2013/MG693173 ^3^
Mon-25	IV	372 nt	MZ382587	97.04% with Cyclovirus TN2/Human/Tunisia/2005/GQ404904
Mon-29 ^3^	I	358 nt	MZ382571	77.65% with Bat circovirus isolate C072/Bat/China/2015/KX834490 ^3^
Mon-32 ^3^	IV	363 nt	MZ382572	97.25% with Cyclovirus TN2/Human/Tunisia/2005/GQ404904 ^3^
Mon-33	I	327 nt	MZ382588	79.20% with Bat circovirus isolate C072/Bat/China/2015/KX834490
Mon-36	VI	281 nt	MZ382589	82.21% with Bat cyclovirus isolate CyV-LimbeP14/Bat/Cameroon/2013/MG693173
Mon-37	IV	374 nt	MZ382590	96.79% with Cyclovirus TN2/Human/Tunisia/2005/GQ404904
Mon-39	IV	350 nt	MZ382591	96.86% with Cyclovirus TN2/Human/Tunisia/2005/GQ404904
Mon-41	IV	354 nt	MZ382592	97.18% with Cyclovirus TN2/Human/Tunisia/2005/GQ404904
Mon-44	IV	357 nt	MZ382593	97.48% with Cyclovirus TN2/Human/Tunisia/2005/GQ404904
Mon-45	VI	275 nt	MZ382594	81.45% with Bat cyclovirus isolate CyV-LimbeP14/Bat/Cameroon/2013/MG693173
Mon-56	IV	366 nt	MZ382595	97.27% with Cyclovirus TN2/Human/Tunisia/2005/GQ404904
Mon-58 ^3^	VII	428 nt	MZ382575	91.84% with Human cyclovirus VS5700009/Human/Malawi/2010-2011/KC771281 ^3^
Mon-59	II	472 nt	MZ382596	98.31% with Pacific flying fox associated cyclovirus-3/Bat/Tonga/2015/KT732789
Mon-60 ^3^	VII	428 nt	MZ382576	91.84% with Human cyclovirus VS5700009/Human/Malawi/2010-2011/KC771281 ^3^
Mon-61	IV	354 nt	MZ382597	96.61% with Cyclovirus TN2/Human/Tunisia/2005/GQ404904
Mon-62 ^3^	VII	428 nt	MZ382577	92.07% with Human cyclovirus VS5700009/Human/Malawi/2010-2011/KC771281 ^3^
Mon-66 ^3^	VIII	370 nt	MZ382578	63.37% with Uncultured virus clone CG263/Environmental sample/USA/2015/KY487932 ^3^
Mon-71	IV	284 nt	MZ382598	97.18% with Cyclovirus TN2/Human/Tunisia/2005/GQ404904
Mon-76	IV	374 nt	MZ382599	97.33% with Cyclovirus TN2/Human/Tunisia/2005/GQ404904

^1^ Partial Rep coding sequences were obtained using the primer CV-R2 as described by Li et al. [3]. Short lengths were trimmed from the end regions to obtain high quality nt sequences. ^2^ Pairwise nt sequence identities were determined using the MUSCLE alignment program (https://www.ebi.ac.uk/Tools/msa/muscle/, accessed 23 June 2021) and the ‘align two or more sequences’ option of BLASTN program (https://blast.ncbi.nlm.nih.gov/, accessed on 23 June 2021). ^3^ The viral strain was molecularly characterized for the complete genome. The nt sequence identity shown here is that based on the partial Rep coding sequence.

**Table 2:** Maximum/significant pairwise nucleotide (nt) sequence identities of the complete genomes of mongoose associated circoviruses and cycloviruses between themselves and with those from other animal species.

**Mongoose associated circovirus**	**GenBank accession number**	**Maximum/Significant Pairwise nt Sequence (%) Identities ^1^**	
		**Between mongoose associated circoviruses**	**With circovirus (Strain Name/Detected in Animal Species/Country/Year/GenBank Accession Number) from other animal species**
Mon-1	MZ382570	99.60% with Mon-29	67.40% with Bat circovirus isolate BtPspp.-CV/GD2012/Bat/China/2012/KJ641716 66.90% with Bat associated circovirus 10, isolate HK02976/Bat/Japan/2013/LC456717
Mon-29	MZ382571	99.60% with Mon-1	67.20% with Bat circovirus isolate BtPspp.-CV/GD2012/Bat/China/2012/KJ641716 66.60% with Bat associated circovirus 10, isolate HK02976/Bat/Japan/2013/LC456717
Mon-66	MZ382578	58.60% with Mon-29 58.50% with Mon-1	60.70% with Porcine circovirus 2 isolate MZ-5/Pig/India/2013/LC004750 60.50% with Porcine circovirus 2 strain MLP-25/Pig/India/2016/MH509731
**Mongoose associated cyclovirus**		**Between mongoose associated** **cycloviruses**	**With cyclovirus (Strain name/Detected in animal species/Country/Year/GenBank accession number) from other animal species**
Mon-20	MZ382573	100% between Mon-20 and Mon-24	72.10% with Bat cyclovirus isolate CyV-LysokaP4/Bat/Cameroon/2013/MG693174 71.50% with Bat cyclovirus isolate CyV-LimbeP14/Bat/Cameroon/2013/MG693173
Mon-24	MZ382574		
Mon-32	MZ382572	65.70% with Mon-20 and Mon-24	77.30% with Cyclovirus isolate PKgoat11/Goat/Pakistan/2009/HQ738636 75.30% with Cyclovirus isolate PK5006/Human/Pakistan/2007/GQ404844
Mon-58	MZ382575	99.30% with Mon-60 and Mon-62	80.20% with Human cyclovirus isolate VS570000/Human/Malawi/2010-2011/KC771281 80.20% with Pacific flying fox associated cyclovirus-3 isolate Tbat_H_103923/Bat/Tonga/2015/KT732788
Mon-60	MZ382576	99.50% with Mon-62 99.30% with Mon-58	79.90% with Human cyclovirus isolate VS570000/Human/Malawi/2010-2011/KC771281 79.90% with Pacific flying fox associated cyclovirus-3 isolate Tbat_H_103923/Bat/Tonga/2015/KT732788
Mon-62	MZ382577	99.50% with Mon-60 99.30% with Mon-58	80.00% with Pacific flying fox associated cyclovirus-3 isolate Tbat_H_103923/Bat/Tonga/2015/KT732788 79.90% with Human cyclovirus isolate VS570000/Human/Malawi/2010-2011/KC771281

^1^ The pairwise nt sequence (%) identities were determined using the Sequence Demarcation Tool Version 1.2 (SDTv1.2) with the MUSCLE alignment algorithm, as described previously [2].

**Table 3:** Maximum deduced amino acid (aa) sequence identities of the putative replication associated proteins (Rep) of mongoose associated circoviruses and cycloviruses between themselves and with those from other animal species.

**Mongoose associated circovirus**	**Maximum/Significant Pairwise Deduced aa Sequence (%) Identities ^1^**	
	**Between mongoose** **associated circoviruses**	**With circovirus/CRESS DNA Virus (Strain Name/Detected in Animal Species/Country/Year/GenBank Accession Number) from other animal species**
Mon-1	99.70% with Mon-29	71.50% with Bat associated circovirus 10, isolate HK02976/Bat/Japan/2013/LC456717
Mon-29	99.70% with Mon-1	71.20% with Bat associated circovirus 10, isolate HK02976/Bat/Japan/2013/LC456717
Mon-66	40.00% with Mon-1 39.60% with Mon-29	51.20% with Syrmaticus reevesii CRESS-DNA-virus sp. isolate phe68cre9/Wild bird/China/2018/MW182878
**Mongoose associated cyclovirus**	**Between mongoose** **associated cycloviruses**	**With cyclovirus (Strain name/Detected in animal species/Country/Year/GenBank accession number) from other animal species**
Mon-20	100% between Mon-20 and Mon-24	78.30% with Bat cyclovirus isolate CyV-LysokaP4/Bat/Cameroon/2013/MG693174 and Bat cyclovirus isolate CyV-LimbeP14/Bat/Cameroon/2013/MG693173
Mon-24		
Mon-32	62.80% with Mon-20 and Mon-24	87.10% with Cyclovirus isolate PKgoat11/Goat/Pakistan/2009/HQ738636
Mon-58	100% between Mon-58, Mon-60, and Mon-62	92.40% with Human cyclovirus isolate VS570000/Human/Malawi/2010-2011/KC771281 88.40% with Pacific flying fox associated cyclovirus-3 isolate Tbat_H_103923/Bat/Tonga/2015/KT732788
Mon-60		
Mon-62		

^1^ The pairwise deduced aa sequence (%) identities were determined using the Sequence Demarcation Tool Version 1.2 (SDTv1.2) with the MUSCLE alignment algorithm [2].

**Table 4:** Maximum deduced amino acid (aa) sequence identities of the putative capsid proteins (Cp) of mongoose associated circoviruses and cycloviruses between themselves and with those from other animal species, or environmental samples.

**Mongoose associated circovirus**	**Maximum/Significant Pairwise Deduced aa Sequence (%) Identities ^1^**	
	**Between mongoose associated circoviruses**	**With circovirus/CRESS DNA Virus (Strain Name/Detected in Animal Species/Country/Year/GenBank Accession Number) from other animal species, or environmental samples**
Mon-1	100% between Mon-1 and Mon-29	42.90% with Bat associated circovirus 10, isolate HK02976/Bat/Japan/2013/LC456717
Mon-29		
Mon-66	24.40% with Mon-1 and Mon-29	44.40% with Uncultured virus clone CG83/Wastewater/USA/2015/KY487977
**Mongoose associated cyclovirus**	**Between mongoose associated** **cycloviruses**	**With cyclovirus (Strain name/Detected in animal species/Country/Year/GenBank accession number) from other animal species**
Mon-20	100% between Mon-20 and Mon-24	59.30% with Bat cyclovirus isolate CyV-LysokaP4/Bat/Cameroon/2013/MG693174 and Bat cyclovirus isolate CyV-LimbeP14/Bat/Cameroon/2013/MG693173
Mon-24		
Mon-32	35.90% with Mon-20 and Mon-24	51.60% with Cyclovirus isolate PK5006/Human/Pakistan/2007/GQ404844
Mon-58	100% with Mon-62	56.10% with Pacific flying fox associated cyclovirus-3 isolate Tbat_H_103923/Bat/Tonga/2015/KT732788
Mon-60	99.6% with Mon-58 and Mon-62	56.60% with Pacific flying fox associated cyclovirus-3 isolate Tbat_H_103923/Bat/Tonga/2015/KT732788
Mon-62	100% with Mon-58	56.10% with Pacific flying fox associated cyclovirus-3 isolate Tbat_H_103923/Bat/Tonga/2015/KT732788

^1^ The pairwise deduced aa sequence (%) identities were determined using the Sequence Demarcation Tool Version 1.2 (SDTv1.2) with the MUSCLE alignment algorithm [2].

## Supplementary Materials

The following are available online at https://www.mdpi.com/article/10.3390/v13091700/s1, Supplementary material S1: Additional primers used in inverse nested PCRs to amplify the complete genomes of mongoose associated circoviruses and cycloviruses. Supplementary material S2: The presence of the arginine rich region in amino terminus of putative capsid proteins of mongoose associated circoviruses (Mon-1 and -29) and cycloviruses (Mon-20, -24, -32, -58, -60 and -62). Supplementary material S3: Two-dimensional graphical representation of pairwise nucleotide sequence (%) identities between the complete genomes of circoviruses detected in various animal species. Supplementary material S4: Two-dimensional graphical representation of pairwise nucleotide sequence (%) identities between the complete genomes of cycloviruses detected in various animal species. Supplementary material S5: Phylogenetic analysis of the putative replication-associated proteins (Rep) of mongoose associated circoviruses (A), and cycloviruses (B), Supplementary material S6: Identification of inter-species recombination event involving mongoose associated cyclovirus strain Mon-32, as determined by the Recombination Detection Program (RDP v.4.101). Supplementary material S7: BLASTN (with coding sequence (CDS) feature) analysis showing the alignment of the putative replication associated protein (Rep) CDS (shown with brown line) of mongoose associated cyclovirus Mon-58 (Query sequence, Rep CDS: join nt 1771-nt 1611, nt 1512-nt 840) with that of Pacific flying fox associated cyclovirus-3 isolate Tbat_H_103923 (GenBank accession number KT732788) (Subject (Sbjct) sequence, Rep CDS: join nt 1788-nt 1628, nt 1515-nt 843).
