# Supplementary material for: Detection and Complete Genome Analysis of Circoviruses and Cycloviruses in the Small Indian Mongoose (Urva auropunctata): Identification of Novel Species
Source: Viruses. 2021 Aug 27;13(9):1700. doi: 10.3390/v13091700 (PMC8471302; doi:10.3390/v13091700)

Supplementary material S7. BLASTN (with coding sequence (CDS) feature) analysis showing the alignment of the putative replication associated protein (Rep) CDS (shown with brown line) of mongoose associated cyclovirus strain Mon-58 (Query sequence, Rep CDS: join nt 1771-nt 1611, nt 1512-nt 840) with that of Pacific flying fox associated cyclovirus-3 isolate Tbat_H_103923 (GenBank accession number KT732788) (Subject (Sbjct) sequence, Rep CDS: join nt 1788-nt 1628, nt 1515-nt 843). The putative intron with the canonical splice donor site (GT) and splice acceptor site (AG) (CA and TC, respectively, in the reverse complemented sequence) is shown with a red line. The 3'- end region of the putative capsid protein (Cp) CDS is shown with a blue line. The stop codons are highlighted with asterisks '*'.

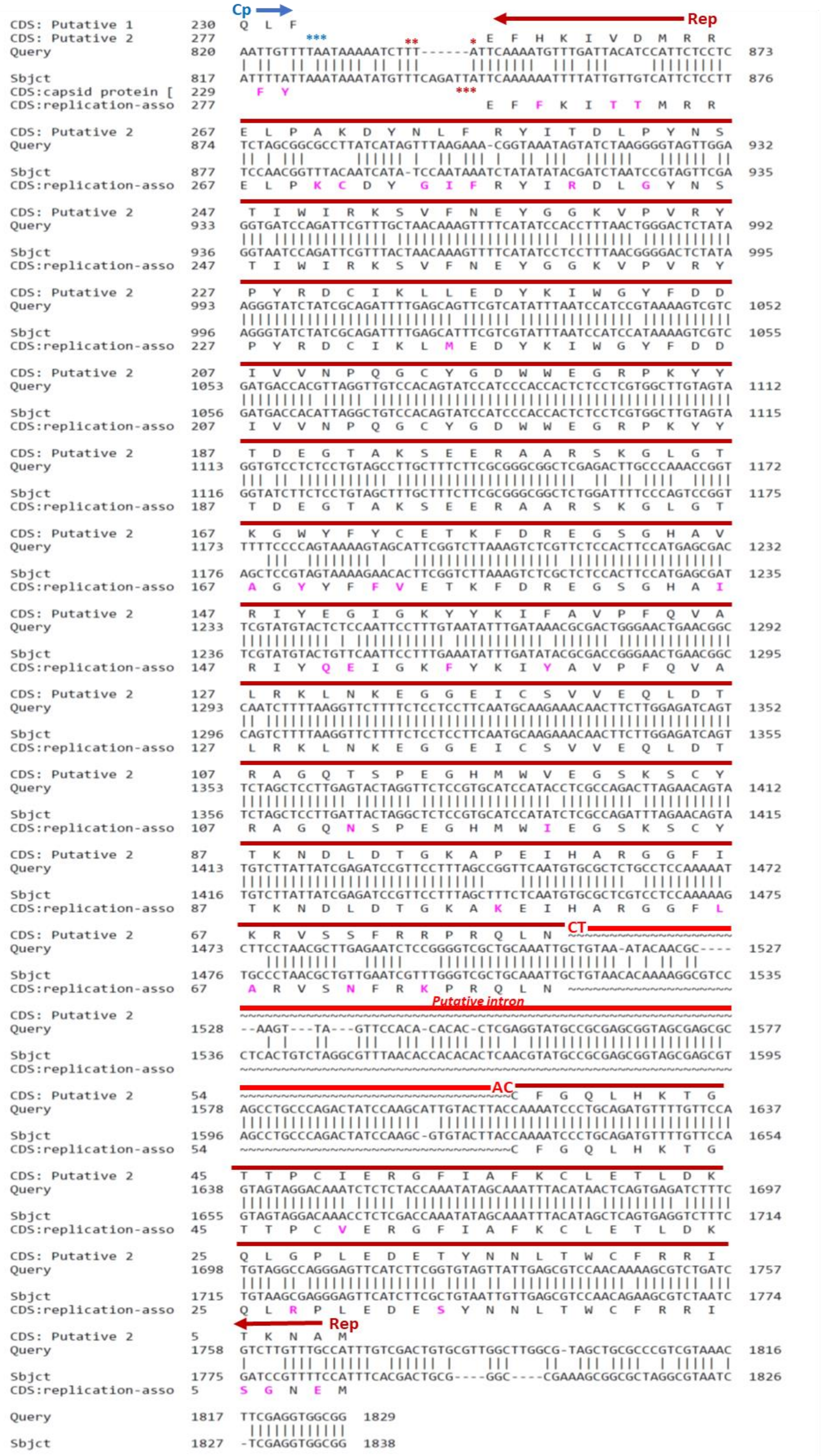

Supplement: Supplementary file 1 [file Supplementary material S7.pdf › Supplementary material S7.pdf]
