# Supplementary material for: Detection and Complete Genome Analysis of Circoviruses and Cycloviruses in the Small Indian Mongoose (Urva auropunctata): Identification of Novel Species
Source: Viruses. 2021 Aug 27;13(9):1700. doi: 10.3390/v13091700 (PMC8471302; doi:10.3390/v13091700)
Supplement: Supplementary file 1 [file viruses-13-01700-s001.zip › Supplementary material S1.pdf]

Supplementary material S1. Additional primers used in inverse nested PCRs to amplify the complete genomes of mongoose associated circoviruses and cycloviruses.

**Mongoose associated circovirus**	**Primer name**	**Primer sequence (5'-3')**	**Primer position**
**Mon-1 and -29**	Mon-1-Circo-F1	ACC CGG AAG TCT ACA TCC GGC ATG [1]	nt 590-nt 613 [2]
	Mon-1-Circo-R1	CTG GCG CGA GGG ACA TCC AAT CTC [1]	nt 510-nt 487 [2]
	Mon-1-Circo-Nested PCR-F2	ACT ACG TGA ATG CTG CTG GAC TGG [1]	nt 629-nt 652 [2]
	Mon-1-Circo-Nested PCR-R2	GAT TCT CCA GAT CCG TCC CCC TCG [1]	nt 454-nt 431 [2]
**Mon-66**	Mon-66-Circo-F1	CGA CCT GCT AGC AAT GAA GAA GGC [3]	nt 468-nt 491 [4]
	Mon-66-Circo-R1	GTA GGT CCA TGG TCC GTC CTG TC [3]	nt 435-nt 413 [4]
	Mon-66-Circo-Nested PCR-F2	GAC GAA GGA CAG AGC GAG AAG G [3]	nt 496-nt 517 [4]
	Mon-66-Circo-Nested PCR-R2	TGT CTT CTT TGC TGC AGT AGG CG [3]	nt 409-nt 387 [4]
**Mongoose associated cyclovirus**			
**Mon-20 and -24**	Mon-24-Cyclo-F1	GGC AGG CAC GTG GTT TGA ACA G [5]	nt 456-nt 477 [6]
	Mon-24-Cyclo-R1	TGC CTT CTC AAT ATG GAT TGC GTC [5]	nt 417-nt 394 [6]
	Mon-24-Cyclo-Nested PCR-F2	ATT TGG AGT CCG TCG TAT CTG CTA [5]	nt 509-nt 532 [6]
	Mon-24-Cyclo-Nested PCR-R2	TTG ATG GCA CTG AAG CGC ATG G [5]	nt 380-nt359 [6]
**Mon-32**	Mon-32-Cyclo-F1	ACA ACG CTA CGA GAT ATT GCC AC [7]	nt 385-nt 407 [8]
	Mon-32-Cyclo-R1	CGT CGG ATC CAT TTG CCT TCT C [7]	nt 268-nt 247 [8]
	Mon-32-Cyclo-Nested PCR-F2	CAT ACG CTA TTT CCG TGG CAT CC [7]	nt 426-nt 448 [8]
	Mon-32-Cyclo-Nested PCR-R2	GCT TCT TGA TGG TAC TGT AGC GTG [7]	nt 223-nt 200 [8]
**Mon-58, -60 and -62**	Mon-58-Cyclo-F1	CTA AGT CTG GCG AGG TAT GGA TGC [9]	nt 427-nt 450 [10]
	Mon-58-Cyclo-R1	GTT CCT TTA GCC GGT TCA ATG TGC [9]	nt 400-nt 377 [10]
	Mon-58-Cyclo-Nested PCR-F2	CGG AGA ACC TAG TAC TCA AGG AGC [9]	nt 452-nt 475 [10]
	Mon-58-Cyclo-Nested PCR-R2	TCC TAA CGC TTG AGA ATC TCC GGG [9]	nt 357-nt 334 [10]

The primer sequences were designed manually following multiple alignment/from the partial Rep-encoding ORF sequence/s of Mon-1 and -29 [1], Mon-66 [3], Mon-20 and -24 [5], Mon-32 [7], and Mon-58, -60 and -62 [9]. The partial Rep-encoding ORF (~400 bp) of the mongoose associated circoviruses and cycloviruses were amplified by nested PCR assays using pan-*rep* primers (CV-F1, CV-R1, CV-F2 and CV-R2) as described previously [3].

Nucleotide (nt) position corresponds to that of the complete genome sequence of mongoose associated circovirus Mon-1 and -29 [2] and Mon-66 [4], and reverse complement of complete genome sequence of mongoose associated cyclovirus Mon-20 and -24 [6], Mon-32 [8], and Mon-58, -60 and -62 [10]. Following ICTV guidelines [1, 2], the first nt of the nonanucleotide motif was considered as 'position one' of the Mon sequences.
