# Supplementary material for: Detection and Complete Genome Analysis of Circoviruses and Cycloviruses in the Small Indian Mongoose (Urva auropunctata): Identification of Novel Species
Source: Viruses. 2021 Aug 27;13(9):1700. doi: 10.3390/v13091700 (PMC8471302; doi:10.3390/v13091700)
Supplement: Supplementary file 1 [file viruses-13-01700-s001.zip › Supplementary material S2.pdf]

Supplementary material S2. The presence of the arginine rich region in amino terminus of putative capsid proteins of mongoose associated circoviruses (Mon-1 and -29) and cycloviruses (Mon-20, -24, -32, -58, -60 and -62). A '-' denotes absence of an amino acid (aa) residue at that position. Numbers to the right indicate the positions of the aa for respective sequences.

```
Mon-1           -------MSRRYRLRRLRPRRSYVRRRHYRRRRYWRRP------YIRR--PRAGAYFIQR 45
Mon-29          -------MSRRYRLRRLRPRRSYVRRRHYRRRRYWRRP------YIRR--PRAGAYFIQR 45
Mon-66          ---MNQYPQKRKGYTVLNNDYKRRRLNQQRENAFWNQVGAMSDMYQSRGNFRTQAAFKKE 57
Mon-20          -------------MRRLRR-VRRAYRRLRRRGSKFRRR------FRRR--NRAS--LACK 36
Mon-24          -------------MRRLRR-VRRAYRRLRRRGSKFRRR------FRRR--NRAS--LACK 36
Mon-32          -----------MALRRVRRYFKRPLRRVHRRRRMFRRR------YTKR--SRSTPTLACK 41
Mon-58          MAFRRRIVRRRAPVRTRRGLFRRRARPFTRRRLALRRA------RARK--ASQRFTLHAR 52
Mon-60          MAFRRRIVRRRAPVRTRRGLFRRRARPFTRRRLALRRA------RARK--ASQRFTLHAR 52
Mon-62          MAFRRRIVRRRAPVRTRRGLFRRRARPFTRRRLALRRA------RARK--ASQRFTLHAR 52
```
