# Supplementary material for: Detection and Complete Genome Analysis of Circoviruses and Cycloviruses in the Small Indian Mongoose (Urva auropunctata): Identification of Novel Species
Source: Viruses. 2021 Aug 27;13(9):1700. doi: 10.3390/v13091700 (PMC8471302; doi:10.3390/v13091700)

**Supplementary material S3.** Two-dimensional graphical representation of pairwise nucleotide sequence (%) identities between the complete genomes of circoviruses detected in various animal species. The plot was constructed using the Sequence Demarcation Tool Version 1.2 (SDTv1.2) with the MUSCLE alignment algorithm , as described previously [2]. The mongoose associated circoviruses are highlighted with asterisks ‘*’.

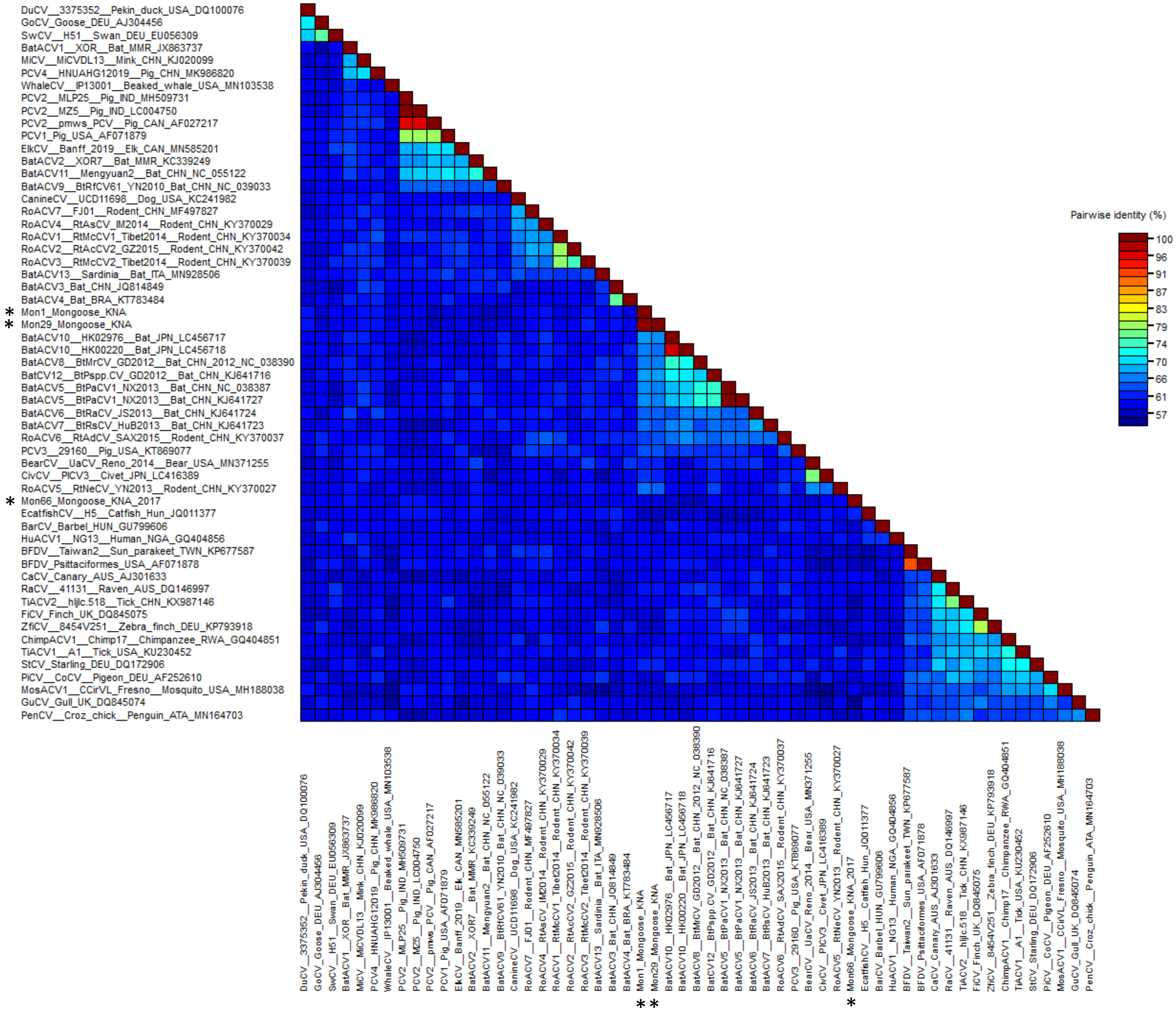

Supplement: Supplementary file 1 [file viruses-13-01700-s001.zip › Supplementary material S3.pdf]
