# Supplementary material for: Detection and Complete Genome Analysis of Circoviruses and Cycloviruses in the Small Indian Mongoose (Urva auropunctata): Identification of Novel Species
Source: Viruses. 2021 Aug 27;13(9):1700. doi: 10.3390/v13091700 (PMC8471302; doi:10.3390/v13091700)

Supplementary material S4. Two-dimensional graphical representation of pairwise nucleotide sequence (%) identities between the complete genomes of cycloviruses detected in various animal species. The plot was constructed using the Sequence Demarcation Tool Version 1.2 (SDTv1.2) with the MUSCLE alignment algorithm, as described previously [2]. The mongoose associated cycloviruses are highlighted with asterisks '*'.

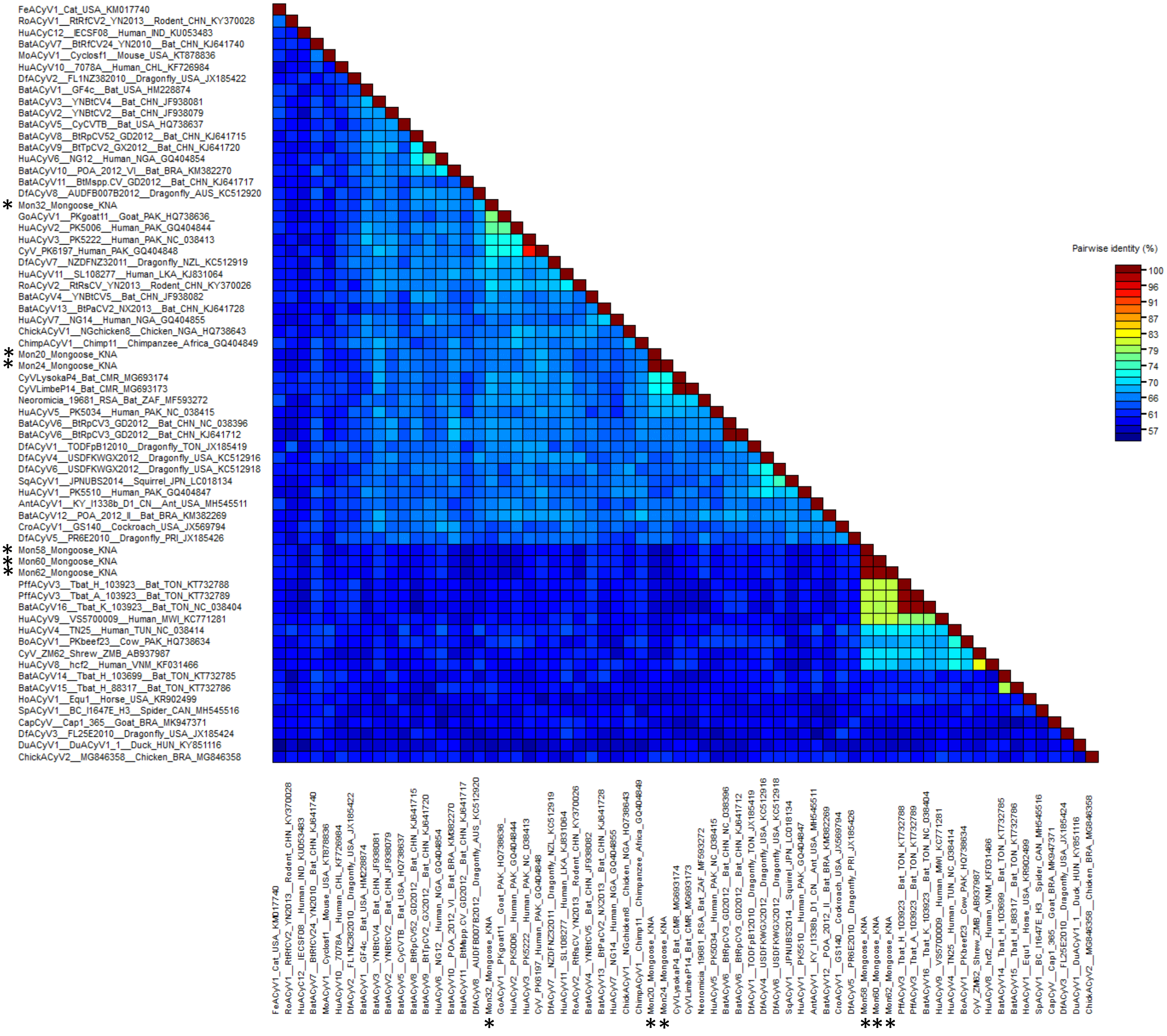

Supplement: Supplementary file 1 [file viruses-13-01700-s001.zip › Supplementary material S4.pdf]
