# Supplementary material for: Detection and Complete Genome Analysis of Circoviruses and Cycloviruses in the Small Indian Mongoose (Urva auropunctata): Identification of Novel Species
Source: Viruses. 2021 Aug 27;13(9):1700. doi: 10.3390/v13091700 (PMC8471302; doi:10.3390/v13091700)

Supplementary material S5: Phylogenetic analysis of the putative replication-associated proteins (Rep) of mongoose associated circoviruses **(A)**, and cycloviruses **(B)**.

(A) Phylogenetic analysis of the putative replication-associated proteins (Rep) of mongoose associated circoviruses (Mon-1, -29 and -66) with those of other circoviruses. The virus name/source (detected in animal species)/country are shown for the Mon sequences, while the species, or virus name (isolate)/source (detected in animal species)/country/GenBank accession number have been mentioned for the other circovirus sequences. The tree was constructed by the maximum likelihood (ML) method, with the LG model of substitution and 1000 bootstrap replicates. Cyclovirus putative Rep sequences were used as the outgroup. Scale bar, 0.5 substitutions per amino acid. Bootstrap values of <80 is not shown. Mon-1 and -29 are shown with black circles, whilst Mon-66 is highlighted with a red circle. BarCV: barbel circovirus; BatACV: bat associated circovirus; BFDV: beak and feather disease virus; CaCV: canary circovirus; ChimpACV: chimpanzee associated circovirus; CivCV: civet circovirus; CV: circovirus; DuCV: duck circovirus; EcatfishCV: European catfish circovirus; ElkCV: elk circovirus; FiCV: finch circovirus; GoCV: goose circovirus; GuCV: gull circovirus; HuACV: human associated circovirus; MiCV: mink circovirus; MosACV: mosquito associated circovirus; PenCV: penguin circovirus; PCV: porcine circovirus; PiCV: pigeon circovirus; RaCV: raven circovirus; RoACV: rodent associated circovirus; StCV: starling circovirus; SwCV: swan circovirus; TiACV: tick associated circovirus; ZfiCV: zebra fish circovirus.

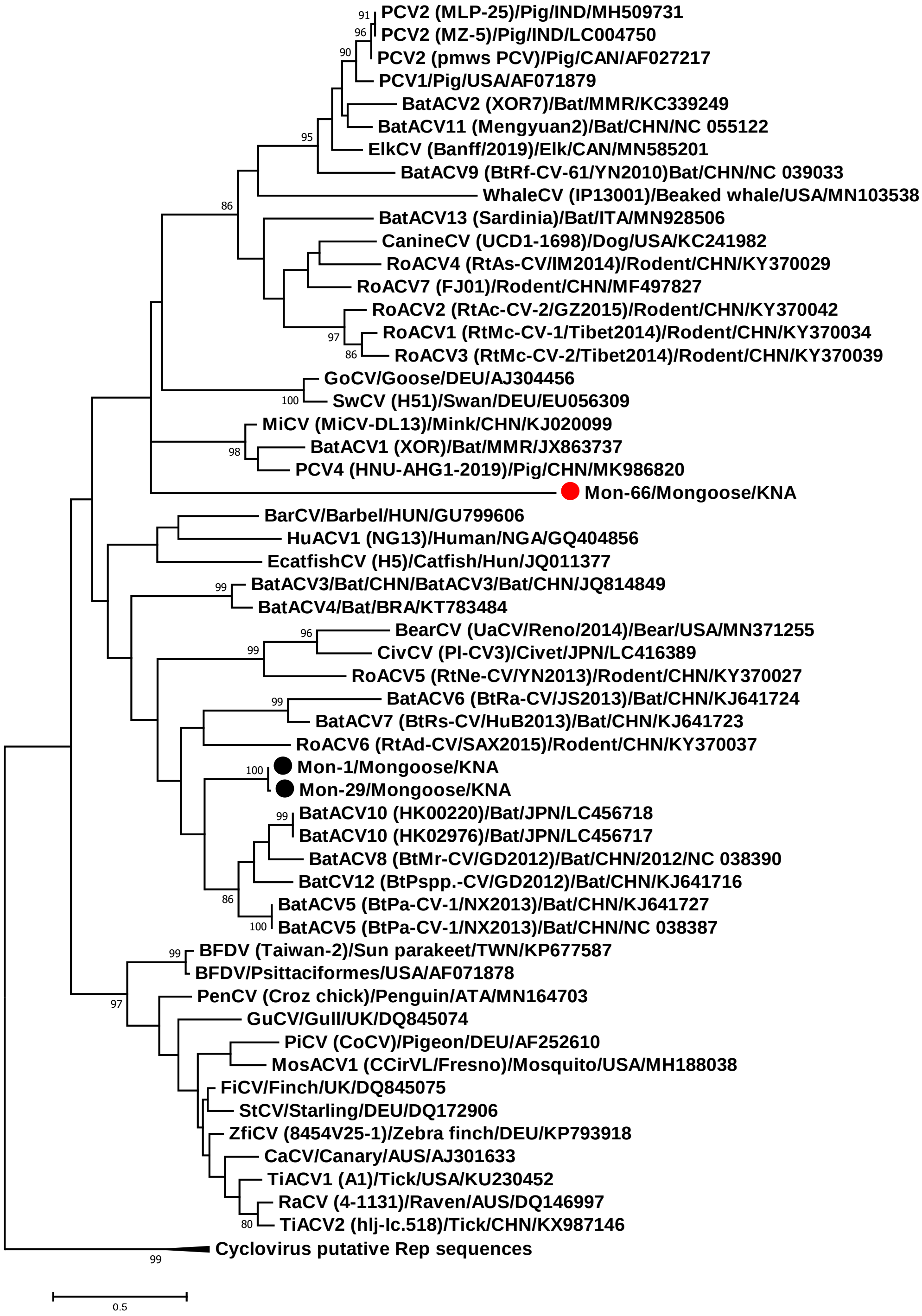

Supplementary material S5: Phylogenetic analysis of the putative replication-associated proteins (Rep) of mongoose associated circoviruses **(A)**, and cycloviruses **(B)**.

(B) Phylogenetic analysis of the putative replication-associated proteins (Rep) of mongoose associated cycloviruses (Mon-20, -24, -32, -58, -60 and -62) with those of other cycloviruses. The virus name/source (detected in animal species)/country are shown for the Mon sequences, while the species, or virus name (isolate)/source (detected in animal species)/country/GenBank accession number have been mentioned for the other cyclovirus sequences. The tree was constructed by the maximum likelihood (ML) method, with the LG model of substitution and 1000 bootstrap replicates. Circovirus putative Rep sequences were used as the outgroup. Scale bar, 0.5 substitutions per amino acid. Bootstrap values of <80 are not shown. Mon-20 and -24 are shown with pink circles, Mon-32 is highlighted with a green circle, whilst Mon-58, -60 and -62 are indicated with brown circles. AntACyV: ant associated cylcovirus; BatACyV: bat associated cyclovirus; BoACyV: bovine associated cyclovirus; ChickACyV: chicken associated cyclovirus; ChimpACyV: chimpanzee associated cyclovirus; CroACyV: cockroach associated cyclovirus; CyV: cyclovirus; DfACyV: dragonfly associated cycovirus; DuACyV: duck associated cyclovirus; FeACyV: feline associated cyclovirus; GoACyV: goat associated cyclovirus; HoACyV: horse associated cyclovirus; HuACyV: human associated cyclovirus; MoACyV: mouse associated cyclovirus; PffACyV: Pacific flying fox associated cyclovirus; RoACyV: rodent associated cyclovirus; SpACyV: spider associated cyclovirus; SqACyV: squirrel associated cyclovirus.

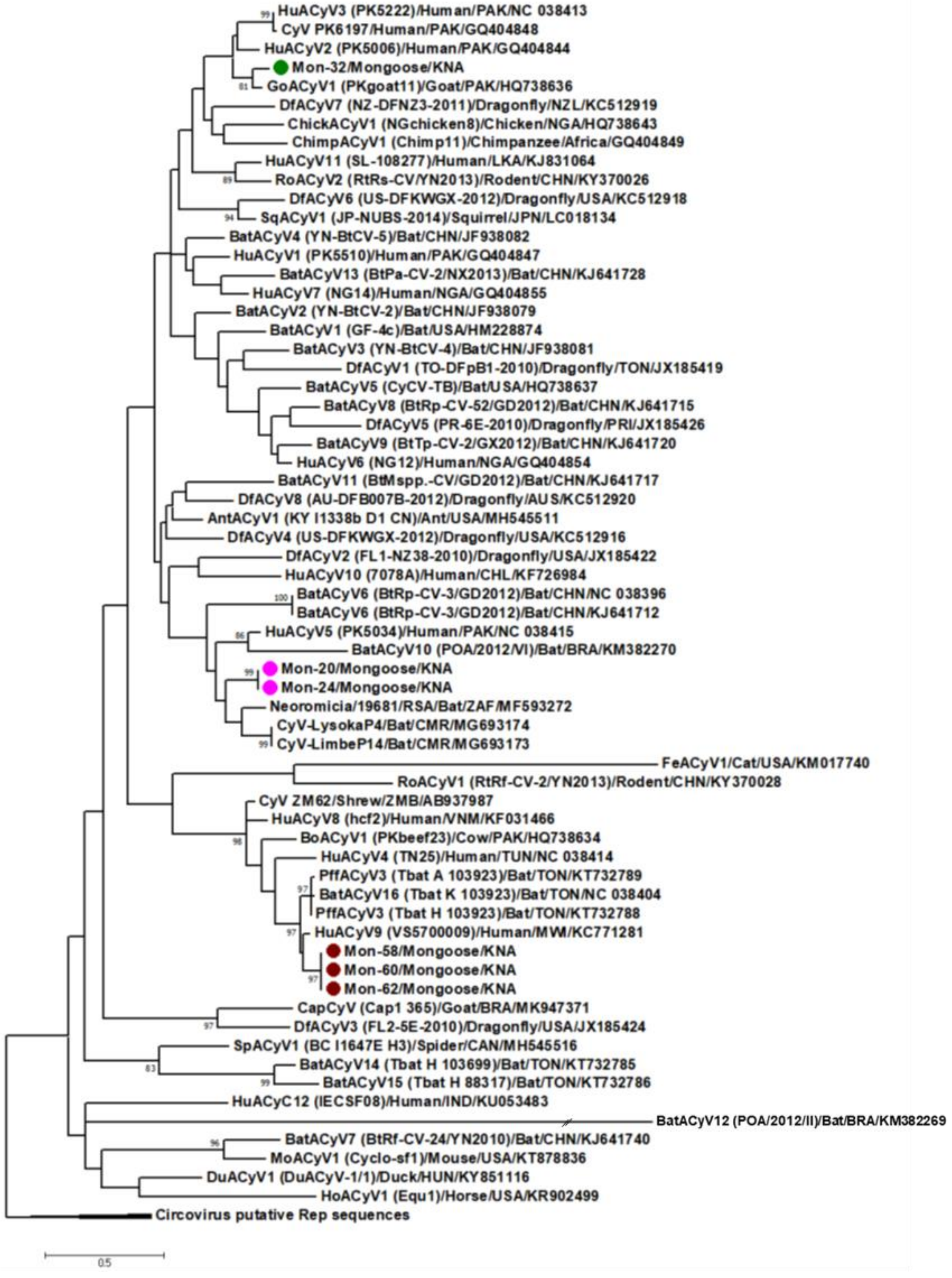

Supplement: Supplementary file 1 [file viruses-13-01700-s001.zip › Supplementary material S5.pdf]
