# Supplementary material for: Detection and Complete Genome Analysis of Circoviruses and Cycloviruses in the Small Indian Mongoose (Urva auropunctata): Identification of Novel Species
Source: Viruses. 2021 Aug 27;13(9):1700. doi: 10.3390/v13091700 (PMC8471302; doi:10.3390/v13091700)
Supplement: Supplementary file 1 [file viruses-13-01700-s001.zip › Supplementary material S6.pdf]

Supplementary material S6. Identification of inter-species recombination event involving mongoose associated cyclovirus strain Mon-32, as determined by the Recombination Detection Program (RDP v.4.101). A sequence was identified as a recombinant if it was detected by more than two methods with a highest acceptable *p-value* of $p < 0.01$ with Bonferroni's correction.

Recombinant: Goat associated cyclovirus 1 (isolate PKgoat11), GenBank accession number HQ738636
Major parent: Unknown
Minor parent: Mongoose associated cyclovirus strain Mon-32
Recombinant region: nt 1174-nt 45

Confirmation table generated by RDP v.4.101 program.

Method	***Average p-value***
RDP	4.973×10^{-15}
GENECONV	1.813×10^{-12}
BootScan	8.837×10^{-18}
MaxChi	7.541×10^{-16}
Chimaera	2.776×10^{-13}
SiScan	5.826×10^{-26}
3Seq	2.226×10^{-05}
